# The influence of flap design on patients’ experiencing pain, swelling, and trismus after mandibular third molar surgery: a scoping systematic review

**DOI:** 10.1590/1678-7757-2020-0932

**Published:** 2021-06-04

**Authors:** Gennaro DE MARCO, Alessandro LANZA, Corina M. CRISTACHE, Estefani B. CAPCHA, Karen I. ESPINOZA, Rosario RULLO, Rolando VERNAL, Emilio A. CAFFERATA, Fabrizio DI FRANCESCO

**Affiliations:** 1 Campania University Luigi Vanvitelli Multidisciplinary Department of Medical, Surgical and Dental sciences Naples Italy Campania University Luigi Vanvitelli, Multidisciplinary Department of Medical, Surgical and Dental sciences, Naples, Italy.; 2 Carol Davila University of Medicine and Pharmacy Faculty of Midwifery and Medical Assisting Department of Dental Techniques Bucharest Romania Carol Davila University of Medicine and Pharmacy, Faculty of Midwifery and Medical Assisting (FMAM), Department of Dental Techniques, Bucharest, Romania.; 3 Universidad Peruana Cayetano Heredia Departamento de Clínica Estomatologica Lima Perú Universidad Peruana Cayetano Heredia, Departamento de Clínica Estomatologica, Lima, Perú.; 4 Universidad de Chile Facultad de Odontología Laboratorio de Biologia Periodontal Santiago Chile Universidad de Chile, Facultad de Odontología, Laboratorio de Biologia Periodontal, Santiago, Chile.; 5 Universidad Científica del Sur Departamento de Periodoncia Escuela de Odontología Lima Perú Universidad Científica del Sur, Departamento de Periodoncia, Escuela de Odontología, Lima, Perú.

**Keywords:** Molar, third, Surgery, oral, Tooth extraction, Surgical flaps, Pain, Swelling, Trismus

## Abstract

Third molar removal surgery usually comes accompanied by postoperative discomfort, which could be influenced by the surgical approach chosen. This scoping systematic review aimed at compiling the available evidence focused on the influence of flap design, including envelope flap (EF), triangular flap (TF), and modified triangular flap (MTF), on postoperative pain, swelling, and trismus, as primary outcome measures, and any result mentioning healing promotion or delay, as secondary outcome measure, after mandibular third molar extraction surgery. An electronic search, complemented by a manual search, of articles published from 1999 to 2020 was conducted in the Medline (PubMed), EMBASE and Web of Science databases including human randomized controlled trials, prospective, and retrospective studies with at least 15 patients. The risk of bias of the included studies was assessed either with the Cochrane’s Risk of Bias tool or with the Newcastle-Ottawa scale. Every step of the review was performed independently and in duplicate. The initial electronic search recovered 2102 articles. After applying the inclusion criteria, 12 articles were included. For patient’s perceived postoperative pain, TF and MTF frequently reported better results than EF. For swelling, the literature is divided, despite a trend favoring EF. For trismus, data showed that its occurrence is mostly associated with the duration of the surgery rather than with the chosen flap. For healing, the limited data is inconclusive. Finally, randomized studies showed a high risk of bias, whereas nonrandomized studies were mostly of good quality and low risk of bias. Although there was no clear consensus regarding the influence of different flap designs for third mandibular molar extraction on postoperative clinical morbidities; the surgeon’s experience, estimated surgical difficulty, molar position and orientation, and surg ery duration should be considered when choosing among the different flap designs.

## Introduction

Impacted teeth refer to a particular anatomical condition in which a tooth fails to erupt within the expected time of physiological development. Third molars are the most common impacted teeth, present in almost 77% of people, and its extraction is the most common oral surgical procedure.^1^ In fact, 33% of the population has at least one impacted third molar, which frequently leads to food retention, caries, pain, edema, and second molar root resorption and, consequently, its surgical extraction.^2,3^ Despite the frequent surgical removal of third molars, the occurrence of accompanying postoperative morbidities is relatively common.^2,3^ The invasive manipulation of soft and hard tissues during tooth extraction involves different factors that can influence the patient’s postoperative course in terms of pain, swelling, trismus, and healing.^4^ In this context, the selection of the surgical access flap can affect the post operative outcomes following third molar surgery, including many complications.

**Figure 1:**
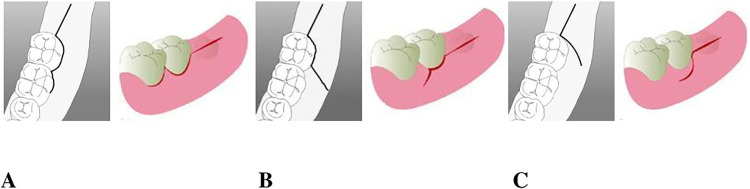
Envelope Flap (A), triangular flap (B) and modified Triangular Flap (C)

Among the available surgical access flaps for third molar surgery, the envelope flap (EF) consists of a linear incision along the top of the alveolar ridge distal to the second mandibular molar, followed by an intrasulcular incision that extends from the distal of the second molar up to, sometimes, the first mandibular molar (Figure 1A), the triangular flap (TF) differs from EF by incorporating a vertical or oblique relief incision in the middle of the second molar vestibular wall, after the intrasulcular incision that reaches 1/3 or 2/3 of its vestibular wall (Figure 1B). Similarly, the modified triangular flap (MTF) starts with an incision from the top of the alveolar crest that reaches the second mandibular molar, but leaves a 2 mm gingival collar around its buccal side, and finishes with a final vertical or oblique relief incision (Figure 1C). Whereas EF uses a single horizontal incision and flap elevation, causing minimal disruption of the vascular supply and facilitating wound closure, TF and MTF use an additional vertical buccal releasing incision, which allows better visibility and accessibility during osteotomy.^5^ Thus, with increasing studies favoring the use of specific flap designs while describing the disadvantages of the other designs, consensus regarding the most harmless flap design for third molar extraction has not yet been achieved.

Although surgeon’s skills and experience often lead to an uneventful third molar removal, this rather invasive intervention is always accompanied by different degrees of postoperative pain, swelling, and trismus. Indeed, individuals that have undergone third molar surgery are frequently impeded to perform their everyday activities. Besides, this surgery is often done on otherwise healthy young people with no history of previous surgeries. Thus, third molar surgical extraction could influence patient’s perceived well-being in different manners, including psychological and social factors affected by pain and discomfort, and consequently on patient’s quality of life. Therefore, this systematic review aimed at analyzing the influence of flap design (intervention), including EF, TF, and MTF (comparison), in mandibular third molar surgery (patient) on the patient’s perceived postoperative pain, swelling, and trismus, considered as primary outcome measures, and any mention of healing promotion or delay, such as the presence of dehiscence or wound gaps, alveolar osteitits, or periodontal health compromise by probing depth augmentation, etc. as secondary outcome measure (outcomes).

## Methodology

### Protocol

The protocol for executing this scoping systematic review, including selection, extraction, and risk of bias assessment phases, was approved *a priori* by all the authors and was constructed following the recommendations of the PRISMA-P checklist, with no posterior amendments.^6^ In addition, for the reporting of this systematic review, the PRISMA Statement was followed accordingly. The formulated focused PICO research question was the following: “In patients (P) that require mandibular third molar surgery (I) is there a difference among performing EP, TF, or MTF (C) regarding patient’s perceived postoperative pain, swelling, trismus, and healing (O)?

### Eligibility criteria

To answer the PICO research question, the inclusion and exclusion criteria were: Randomized controlled trials (RCTs) and nonrandomized prospective or retrospective studies performed in humans, including at least 15 patients treated for third molar extraction, comparing at least two flap designs (EP, TF, or MTF), evaluating at least two patient’s postoperative clinical outcomes, including pain reported using a visual analogue scale (VAS), swelling estimated by measuring the operation area before and after the procedure, trismus estimated by measuring the mouth opening distance before and after the procedure, and any mention of healing promotion or delay, such as the presence of dehiscence or wound gaps, alveolar osteitis, or periodontal health compromise by probing depth augmentation, and published in English. Publication status or grey literature were not considered as exclusion criteria.

### Literature search

A search strategy using the combination of free-text words including: "Mandibular third molar surgery", "mandibular third molar flap design", "envelope flap ", "triangular flap", "modified triangular flap", was performed in the Medline (PubMed), EMBASE, and Web of Science databases up to May 2020. In addition, the search on the included studies references was complemented manually. If data were missing, corresponding authors were reached via e-mail.

### Data selection and extraction

Data selection (F.D and G.D) and extraction (F.D and CM.C) were performed by two authors, independently. First, titles and abstracts were assessed for potential inclusion. Then, full-text articles were evaluated against the inclusion criteria. The inclusion and exclusion of studies were decided by consensus between the two authors in every step of the selection phase. If disagreements occurred, inclusion or exclusion of the studies was consulted with a third author (A.L). Data extraction was performed in a pre-designed sheet by collecting the following data: Authors, study design, number of patients, flap design, the position of the extracted third molars, according to the Pell and Gregory classification,^7^ follow-up period, and patient’s reported postoperative clinical outcomes.

### Risk of bias and quality of the studies assessment

The risk of bias of the included studies was evaluated independently and in duplicate by two reviewers (G.D and F.D). To analyze randomized controlled trials, the Cochrane risk of bias tool, analyzing selection, performance, detection, attrition, reporting, and other biases, was used by assessing the following parameters: random sequence generation, allocation concealment, blinding of the examiner and/or patient, post-operative follow-up and incomplete outcome data.^8^ Moreover, to analyze nonrandomized clinical studies, the Newcastle – Ottawa Scale (NOS) was used.^9^ This scale uses a star system, in which a study is judged based on three broad perspectives: The selection of the study groups (up to 4 stars), the comparability of the groups (up to 2 stars), and exposure or outcome of interest for case-control or cohort studies, respectively (up to 3 stars). Studies that met five or more of the Newcastle – Ottawa Scale criteria were considered as low risk of bias and good quality. Finally, data from the included studies were assessed in a qualitative manner.

## Results

**Figure 2:**
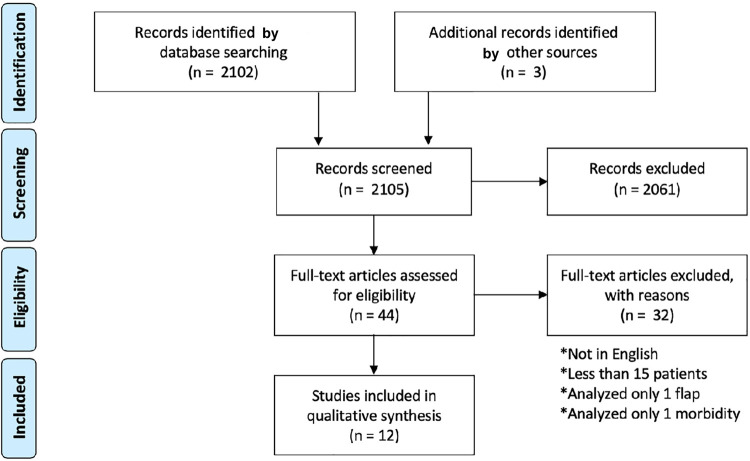
PRISMA Flow chart for the data selection process

The initial search identified 2,102 potential items. After reading the titles and abstracts, 41 articles were selected for full-text revision. Then, the full-text analysis excluded 32 studies that did not evaluate at least two of the examined flaps or at least two of the postoperative clinical outcomes. Finally, nine articles were considered eligible. Subsequently, three articles were added after the manual search, leading to a total of 12 articles included in the review,^10-21^ as shown in the data selection flow chart (Figure 2). The data regarding the number of patients, the examined flaps, and clinical outcomes are shown in Table 1 and Table 2.

**Table 1:** Descriptive summary of included studies

Author	Study Design	Flap	Controls and Follow up	Pell and Gregory Classification
Alqahtani, et al^10^	Retrospective	EF/MTF	1, 3, 7, 8, 15 days and 3 weeks	NR
Mohajerani, et al^11^	RCT	EF/MTF	3, 7 days	I, II/C
Mobilio, et al^12^	RCT	EF/TF	2, 7 days	NR
Rabi, et al^13^	Prospective	E/TF	2, 3, 7 days	NR
Desai, et al^14^	RCT	EF/TF	15 days	NR
Koyuncu, et al^15^	Prospective	EF/MTF	1, 2, 7 days	NR
Baqain, et al^16^	RCT	EF/TF	2, 7, 14 days	NR
Erdogan, et al^17^	Retrospective	EF/TF	3, 7 days	I, II/A, B
Sandhu, et al^18^	Retrospective	EF/MTF	1, 3, 7, 14, 30 days	NR
Kirk, et al^19^	Retrospective	EF/MTF	1, 2, 7 days	NR
Dolanmaz, et al^20^	Prospective	EF/MTF	7 days	NR
Saima, et al^21^	Prospective	EF/TF	2, 7 days	NR

RCT: randomized clinical trial; EF: envelope flap; TF: triangular flap; MTF: modified triangular flap; NR: not reported.

**Table 2:** Reported differences between postoperative occurrence of pain, swelling, trismus and healing after using different access flap designs for third molar surgery in the included studies

Author	Year	Patient Number	EF	TF	MTF	Pain	Swelling	Trismus	Healing
Alqahtani, et al^10^	2017	60	60		60	=	< EF*	ND	ND
Mohajerani, et al^11^	2018	31	28		28	ND	ND	=	> MTF†
Mobilio, et al^12^	2017	25	12	13		=	=	=	ND
Rabi, et al^13^	2017	50	25	25		=	ND	< MTF*	ND
Desai, et al^14^	2014	30	15	15		=	< EF*	ND	=
Koyuncu, et al^15^	2013	80	40		40	< MTF†	< MTF†	ND	ND
Baqain, et al^16^	2012	19	19	19		=	< EF*	< TF*	ND
Erdogan, et al^17^	2011	20	20	20		< TF†	< EF†	=	ND
Sandhu, et al^18^	2010	20	20		20	< MTF*	=	=	> MTF*
Kirk, et al^19^	2007	32	32		32	=	< MTF†	=	ND
Dolanmaz, et al^20^	2013	30	30		30	=	=	ND	ND
Saima, et al^21^	2017	284	142	142		=	=	=	ND

EF: Envelope flap, MTF: modified triangular flap, TF: triangular flap. <: Less postoperative occurrence, >: More postoperatively occurrence; =: No statistical difference reported, †: Statistically not significant trend reported; *: Statistically significant difference reported. ND: Not determined.

### Flap selection effect over postoperative pain

Many authors agree that TF and MTF have better results than EF regarding postoperative pain after third molar surgery;^15,18^ however, these differences are not all statistically significant.^17^ According to Sandhu,et al.^18^ (2010), patients in the EF group experienced significantly more pain as compared to the MTF group (P<0.05). Similarly, Koyuncu and Cetingül^15^ (2013) described that MTF-intervened patients also reported less postoperative pain. Kirk, et al.^19^ (2007), in turn, showed no statistically significant differences between the EF and MTF groups regarding pain.

Although EF is the most commonly used surgical approach for lower third molar removal, the extensive exposition of buccal bone from the adjacent second molar during this procedure has been frequently associated with patients perceiving more pain, when compared with the other less invasive approaches.^15^ This could be also attributed to the incision, the damage to the second molar periodontal tissues, the reflection of the mucoperiosteal flap, and the removal of bone during the procedure. Moreover, the occurrence of wound dehiscences at the distofacial edge of the second molar and the length of the surgical procedure could also lead to a prolonged period of discomfort and pain. Besides, experience of the surgeon, type of impact, administration of preoperative or postoperative corticosteroids, and compliance to postoperative instructions could also affect the pain experienced by the patient.^17,18^ Finally, most articles agreed that pain was the most frequently reported comorbidity, mostly on the immediate days after surgery, and that it decreased continuously over the healing course, regardless of the surgical technique. Since it requires a soft diet and several rest days, it negatively affects patient’s daily routine and, consequently, the patient’s quality of life.^22^

### Flap selection effect over postoperative swelling

Alqahtani, Khaleelahmed and Desai^10^ (2017) compared EF and MTF during third molar surgery, showing significantly better outcomes for the EF group regarding postoperative swelling. Similarly, Baqain, et al.^16^ (2012) reported that patients intervened with EF, when compared with TF, showed significantly less postoperative swelling. On the other hand, Dolanmaz, et al.^20^ (2013) showed no significant differences regarding swelling when comparing patients treated with an EF approach and with an MTF approach. Sandhu, Sandhu and Kaur^18^ (2010) also claimed that there was no difference in postoperative swelling between the patients treated with EF and those treated with MTF. Koyuncu and Cetingül^15^ (2013) and Kirk, et al.^19^ (2007), in turn, reported less swelling among the patients treated with the MTF approach when compared with the EF group; however, these differences were not statistically significant. Despite the considerable trend favoring that the EF approach could lead to less postoperative swelling after third molar extraction, the literature available is divided.

TF and MTF association with increased facial swelling could be explained, at least partly, by the buccal releasing incision, which provokes increased local inflammation and subsequent edema in the buccal tissues.^10,16^ In fact, surgical incisions extension and quantity of bone removal have been associated with the severity of facial swelling. Furthermore, the incidence of facial swelling also depends on the type of third molar impact, the difficulty of extraction operation, and the oral hygiene of the patient. Although many studies have attempted to determine predictive factors and preventive interventions for facial swelling, inconsistency between the results compromises patients’ perception of the quality of the dentist’s service, follow-up, and of their own quality of life.^23^

### Flap effect over postoperative trismus

Erdogan, et al.^17^ (2011) reported that there were no statistically significant differences between the EF and TF groups regarding trismus. Similarly, Sandhu, Sandhu and Kaur^18^ (2010) and Kirk, et al.^19^ (2007) showed no differences in the occurrence of postoperative trismus between patients approached with EF and those approached with MTF. Conversely, Baqain, et al.^16^ (2012) showed a statically significant difference favoring TF over EF group regarding trismus. Nevertheless, Mobilio, et al.^12^ (2017) showed that the duration of surgery, and not the flap design, was associated with the acute postoperative symptoms, including trismus, after lower third molar extraction. Thus, the analyzed data showed that the occurrence of trismus could be associated with the duration of the surgery, although patients treated with TF or MTF flaps presented fewer trismus events.

Traumatic manipulation of tissues during third molar extraction can lead to trismus. Mouth opening length reduction accompanied by a decrease of masticatory muscle activity has been frequently reported after third molar surgery. Indeed, the reduction of muscular activity on the intervened site has been considered as an innate protective and analgesic function to diminish pain. Moreover, direct muscle damage and acute inflammation may provoke adjacent muscle spasms and lead to limited mouth opening.^24^ Finally, trismus-provoked dysphagia is also a frequent undesired effect of third molar surgery, which negatively affects patient’ s quality of life by limiting conventional eating and requiring unpleasant soft or liquid diets.^25^

### Flap effect over tissue healing

Healing is often not reported as a clinical parameter after third molar surgery; however, the few articles analyzing healing showed better healing in patients treated with the MTF approach.^11,14,18^ Mohajerani, et al.^11^ (2018) showed that the application of MTF might lead to a reduction in dry socket incidence and better healing 7 days after lower-impacted third molar surgeries. On the other hand, Desai, et al.^14^ (2014) reported no statistical differences between EF and TF-treated patients in the healing of flap due to presence of gaps, hematoma, sensitivity of adjacent teeth, and dry socket. When considering the initial phases of healing, alveolar osteitis (AO) can be considered as a relatively frequent complication. In this context, Koyuncu and Cetingül^15^ (2013) found no differences between the incidence of AO when using either EF or MTF approaches. Interestingly, Elo, et al.^26^ (2016) proposed a modified approach by incorporating a double-pass single-layered running continuous primary closure to provide a tighter protection of the clot. The modified flap design, which consisted in a sulcular incision starting at the midfacial portion of the second molar and extending distolaterally across the lateral body or ramus of the mandible, resulted in a significantly less risk of developing AO and other complications when compared with both the traditional EF and MTF designs.

Clinical healing delay negatively affects patient’s oral health-related quality of life recovery, and has been associated with symptomatic third molars and surgical difficulty.^27^Besides, surgical extraction of unerupted impacted third molars can damage to the second molar periodontium permanently. However, a recent meta-analysis found variations in second molar probing depth around 1 mm, only during the first three months after surgery, thus having a limited clinical impact.^28^ Moreover, triangular flaps with paramarginal incisions are expected to better preserve second molar periodontal health by leaving an untouched band of keratinized tissue around the second molar.^28^

### Risk of bias and quality of the included studies

Tables 3 and 4 shows the outcomes of the risk of bias assessment of included studies. All four RCTs studies^11,12,14,16^showed a high risk of bias in one or two key domains. One study showed an unclear risk of bias in both allocation concealment and blinding during the result survey,^14^ whereas all studies showed an unclear risk of bias in at least one of them, as shown in Table 4. The scores of the five nonrandomized studies eligible for the NOS ranged from 5 to 8 stars.^10,13,15,17-21^ According to the authors’ definitions, the overall ranking showed no studies with a low risk of bias and that all of them were of good quality.

**Table 3:** Risk of bias and quality assessment of included nonrandomized studies using the Newcastle-Ottawa Scale

Study	Selection	Comparability	Outcome	Total
	max 4 ****	max 2 **	max 3 ***	
Alqahtani, et al^10^	***	*	***	7
Rabi, et al^13^	***	*	**	6
Kovuncu, et al^15^	****	*	***	8
Erdogan, et al^17^	****	*	***	8
Sandhu, et al^18^	****	**	***	9
Kirk, et al^19^	****	*	**	7
Dolanmaz, et al^20^	****	**	**	8
Saima, et al^21^	***	*	*	5
			NOS SCORE	≥5

**Table 4:** Risk of bias assessment of included RCTs using the Cochrane’s Risk of Bias tool

Study	Sequence Generation	Allocation Concealment	Blinding Operators and Participants	Blinding of Results Surveys	Incomplete Outcome Data	Selecting Outcome Reporting	Other Bias
Mohajerani, et al^11^	low	low	high	unclear	low	low	no
Mobilio, et al^12^	low	unclear	high	low	low	low	no
Desai, et al^14^	high	unclear	high	unclear	low	low	no
Baqain, et al^16^	low	low	high	unclear	low	low	no

## Discussion

Although the removal of mandibular third molars is one of the most common surgical procedures, it is often associated with patients experiencing postoperative complications, such as perceived pain, swelling, and trismus, regardless of the surgical approach.^29^ Thus, this review aimed at investigating if the use of different flaps design (EF, TF or MTF) for third molar surgery influenced patients’ perceived postoperative clinical occurrences.

Each of the three analyzed flaps has particular advantages and disadvantages.^30^According to Mohajerani, et al.^11^ (2018), the decrease in surgical complications following third molar surgery is an important issue, which could be achieved by designing an appropriate flap. Different clinical studies have reported the advantages of using TF and MTF approaches,^15,31^ including an increase in the operative visibility of the surgical site, lower incidence of damage to the flap, and better management of intra operative complications, especially for a less experienced surgeon. In the case of the EF approach, its main advantage is less intraoperative bleeding due to the less surgical invasiveness, and to the fewer damage to the periosteum and buccinator muscle.

Patients’ experiencing negative postoperative outcomes could be decreased if surgical decision-making was based on tooth radiographic location and orientation. Despite being dated, the most accredited third molar inclusion classifications are the Classification of Winter from 1926^32^ and Classification of Pell and Gregory from 1933.^7^ Winter classified the impacted teeth according to their angulation in vertical, horizontal, mesioangular, and distoangular.^33^ Alternatively, Pell and Gregory classified the impacted teeth according to their relation with the second molar occlusal plane, in classes A, B, and C, and according to their proximity to the anterior border of the mandibular ramus, in classes I, II, and III.^7^ Different clinical approaches have been recommended depending on tooth position; the MTF is suggested for impacted mandibular third molars in Class 3-Position C, whereas the EF approach is recommended for teeth in Class 1-Position A. However, intermediate classes and positions, i.e. combinations including Class 2 and Position B, are not the most frequently reported.^33^ In these cases, the choice of the access flap is determined by the estimated difficulty of the intervention, by considering the depth of the inclusion and the position of the third mandibular molar. Indeed, based on the preoperative data, the Pederson’s scale was used to define the level of difficulty of all extractions before the surgery, classifying them as easy, moderately difficult, or very difficult.^34^ When Pederson’s scale is easy, the EF approach is chosen and, when preoperative Pederson's scale is moderately difficult or difficult, the use of TF or MTF is preferred.

According to two recent systematic reviews, Lopes da Silva, et al.^35^ (2020) and Glera-Suárez, et al.^36^ (2020), there are no statistically significant differences regarding postoperative clinical morbidities when comparing the use of different access flaps for third mandibular molar surgery in the literature when assessing RCTs. In the context of our study, when assessing intervention and observational studies, substantial heterogeneity was found among the included studies. In fact, their results could be influenced by different parameters, such as patients characteristics (sex and age), intervention features (surgeon experience, surgical materials, and duration of surgery), and outcome measures (pain rating scale, swelling assessment methods, outcomes, and follow-up). These limitations are often found when comparing clinical trials in Dentistry,^37,38^ impeding the performance of an adequate quantitative analysis in systematic reviews, such as the case of our investigation. We suggest that future research should consider the standardization of the outcome measures for evaluating postoperative events, clear patient selection, and similar operator experience. Finally, an estimated surgical difficulty and probability of tissue damage, based on a reliable radiographic tooth position and orientation classification, should be considered to establish a defined surgical protocol for third mandibular molar extraction, with minimal postoperative complications.

## Conclusion

There was no clear consensus among the reviewed studies that a particular flap design for third mandibular molar surgery could have advantages regarding patient’s perceived postoperative clinical morbidities. Cumulative evidence suggest that flap selection association to surgical difficulties is mainly determined by impacted tooth position. In fact, the tissue manipulation performed during flaps, which leads to patient’s discomfort, aims to increase surgical visibility area and further reduce surgical time. Thus, a flap design is chosen based on surgeon’ s experience, molar position and orientation and, finally, these characteristics along with the duration of the surgical procedure, directly affect patients’ postoperative experience.
